# Genome-wide analysis of metallothionein gene family in maize to reveal its role in development and stress resistance to heavy metal

**DOI:** 10.1186/s40659-021-00368-w

**Published:** 2022-01-10

**Authors:** Canhong Gao, Kun Gao, Huixian Yang, Tangdan Ju, Jingyi Zhu, Zailin Tang, Liangxia Zhao, Qingquan Chen

**Affiliations:** School of Agronomy, Anhui Agricultural University, Anhui province, Hefei, 230036 People’s Republic of China

**Keywords:** Maize, Metallothionein gene family, Identification, Expression patterns, Heavy metal stress

## Abstract

**Background:**

Maize (*Zea mays* L.) is a widely cultivated cereal and has been used as an optimum heavy metal phytoremediation crop. Metallothionein (MT) proteins are small, cysteine-rich, proteins that play important roles in plant growth and development, and the regulation of stress response to heavy metals. However, the MT genes for maize have not been fully analyzed so far.

**Methods:**

The putative *ZmMT* genes were identified by HMMER.The heat map of *ZmMT* genes spatial expression analysis was generated by using R with the log^2^ (FPKM + 1).The expression profiles of *ZmMT* genes under three kinds of heavy metal stresses were quantified by using qRT-PCR. The metallothionein proteins was aligned using MAFFT and phylogenetic analysis were constructed by ClustalX 2.1. The protein theoretical molecular weight and pI, subcellular localization, TFs binding sites, were predicted using ProtParam, PSORT, PlantTFDB, respectively.

**Results:**

A total of 9 *ZmMT* genes were identified in the whole genome of maize. The results showed that eight of the nine ZmMT proteins contained one highly conserved metallothio_2 domain, while ZmMT4 contained a Metallothio_PEC domain. All the ZmMT proteins could be classified into three major groups and located on five chromosomes. The *ZmMT* promoters contain a large number of hormone regulatory elements and hormone-related transcription factor binding sites. The *ZmMT* genes exhibited spatiotemporal specific expression patterns in 23 tissues of maize development stages and showed the different expression patterns in response to Cu, Cd, and Pb heavy metal stresses.

**Conclusions:**

We identified the 9 *ZmMT* genes, and explored their conserved motif, tissue expression patterns, evolutionary relationship. The expression profiles of *ZmMT* genes under three kinds of heavy metal stresses (Cu, Cd, Pb) were analyzed. In summary, the expression of *ZmMTs* have poteintial to be regulated by hormones. The specific expression of *ZmMT*s in different tissues of maize and the response to different heavy metal stresses are revealed that the role of MT in plant growth and development, and stress resistance to heavy metals.

**Supplementary Information:**

The online version contains supplementary material available at 10.1186/s40659-021-00368-w.

## Background

Excessive heavy metal ions, such as cadmium (Cd), copper (Cu), lead (Pb) often cause heavy metal stress in plants, resulting in metabolic dysfunction and growth inhibition [1]. Plants have evolved several mechanisms for detoxification of heavy metal, such as efflux of heavy metals from the cell, chelation, and sequestration heavy metal ions through specific transporters or ligands [1–3]. The glutathione (GSH), phytochelatins (PCs), and metallothionein (MT) are three well-known heavy metal-binding ligands in plant cells [4–7]. MT plays an important role in response to heavy metal stress and has been a research hotspot in the field of molecular biology [8].

MT proteins are small (7–8 kDa), cysteine-rich (20–30%) can keep the metal ion homeostasis by binding with heavy metal ions, and protect against heavy metal toxicity by sequestration [9–12]. The first plant MT protein was identified in wheat (EcMT) in 1987 [13], then more MT sequences have been reported in various species [14–34]. There are 7 and 14 *MT* genes in *A. thaliana* and *O. sativa* respectively, which can be classified into four subfamilies (type/class 1, 2, 3 and 4) [28, 33, 34]. The 6 *MT* genes in sugarcane (*Saccharum officinarum* L.) were found, which *ScMT3-1* plays an active role in yeast (*P. pastoris*) response to Cd^2+^ and Cu^2+^ stress[35].Previous investigations showed that plant MT proteins acted as reactive oxygen species (ROS) scavenging enzymes [19, 36–38]. For example, rice *OsMT1a* and *OsMT2b* and cotton (*Gossypium hirsutum*) *GhMT3a* possessed of superoxide- and hydroxyl radical-scavenging activities in vitro [36–38]. Moreover, the lack of *OsMT2b* expression was found to promote epidermal cell death in stems and to accelerate H_2_O_2_-mediated aerenchyma formation in the internodes in rice [39, 40]. In maize, the expression level of *MT1* in the root cortex was found to decrease during aerenchyma formation under waterlogged conditions [41]. These findings suggested that MT proteins had a role in determining the fate of cells in roots during inducible aerenchyma formation.

Maize (*Zea mays* L.) is a widely cultivated cereal and has been widely adopted for phytomanagement of Cd-contaminated soils due to its high biomass production and Cd accumulation capacity [42]. Recently, an increasing number of transcriptome studies screened out a series of candidate genes involved in the responses to heavy metal ions stress in various plant species [43–51]. Most transcriptome studies only provide fundamental information on the pathway involved in the responses to heavy metal ions stresses.

Although the *MT* genes have been studied in several plant species, however, they are rarely reported in maize. In this study, we identified 9 *ZmMT* genes in maize genome-wide, and their conserved motif and tissue expression patterns were analyzed. To understand their evolutionary relationship with other plants, a phylogenetic tree was constructed. Furthermore, the expression profiles of the *ZmMT* genes under three heavy metal stresses (Cu, Cd, Pb) were assessed by using qRT-PCR. The findings of our study will help to understand the roles of *ZmMT* genes in heavy metal ions response and to further identify the functions of this essential gene family in maize.

## Materials and methods

### Plant materials and stress treatments

Seeds of maize inbred line B73 were surface sterilized in 0.5% NaClO for 5 min, washed with distilled water, and then germinated on the filter paper moistened with distilled water and incubated at 26℃ in the dark. Seedlings in identical growing situations were selected, after three days, transplanted into an aerated complete nutrient solutions (Additional file 1: Table S1) and grew in a growth chamber as follows: kept for 3 days with a photoperiod of 14 h light/10 h dark at 26 ℃ and relative humidity of 70% [52]. After that, the maize seedlings were randomly divided into two groups, CK-grown seedlings, grown only in half-strength Hoagland solution were regarded as controls; Cd 200-grown (200 mg/L CdCl_2_·2.5H_2_O), Pb 1000-grown (1000 mg/L, Pb(NO_3_)_2_), Cu 200-grown (200 mg/L, CuCl_2_·2H_2_O) for heavy metal stresses. Both heavy metals (Cd, Pb, Cu)-grown and CK-grown root, stem, leaves of seedling three different tissues were separately sampling at 0 h, 24 h respectively after heavy metal treatment. All samples were harvested from each of the three maize seedlings, and three independent replicates were collected for each sample. Following harvested and immediately frozen in liquid nitrogen and stored at – 80 ℃ for extracting RNA and qRT-PCR analysis.

### Identification of metallothionein genes in maize

The protein sequences of *Zea mays* (Zm, B73_RefGen_v4) were downloaded from Ensembl Plants (http://plants.ensembl.org/index.html). MT protein sequences of *A. thaliana* and *O. sativa* were downloaded from TAIR (https://www.arabidopsis.org/) and RGAP (http://rice.plantbiology.msu.edu/).

HMMER 3.0 software (http://hmmer.janelia.org/) was used to identify the putative *MT* genes by searching MT domain which was made by MT protein sequences of *A. thaliana* and *O. sativa* under default parameters. The putative *MT* genes were annotated by Pfam database. The protein theoretical molecular weight and pI were predicted using ProtParam (http://au.expasy.org/tools). The subcellular localization of *ZmMT* genes was predicted using the PSORT program (https://psort.hgc.jp/).

### Phylogenetic analysis and conserved motif analysis

For the MT proteins phylogenetic analysis, all the MT proteins from *Zea mays*, *A. thaliana* and a outgroup sequence ScMT1 (S.cerevisiae metallothionein, accession No. AAA66061) were aligned using MAFFT software version 7 with L-INS-I [53], then a phylogenetic tree of *Zea mays* and *A. thaliana* MT proteins was constructed using ClustalX 2.1 software with 1000 bootstrap replicates [54]. The online program MEME Version 5.0.5 (http://meme.nbcr.net/meme/) was used to analyze conserved motifs for the 9 *ZmMTs* sequences with parameters as follows: maximum number, 5; site distribution, any number of repetitions; minimum width, 6; and maximum width, 50.

### *ZmMT* genes distribution on Chromosomal and structure analysis

The chromosomal distribution of ZmMT genes was obtained from the Ensembl Plants (http://plants.ensembl.org/index.html), and the location images was drawn with TBtools software (https://github.com/CJ-Chen/TBtools/releases) [55]. Gff3 file of *ZmMT* genes was used for drawing schematic diagram gene structure.

### Calculation of Ks and Ka of *ZmMTs*

Paralogous gene pairs of *ZmMT* genes were identified by Orthofinder v2.2.6 with the BLAST method under default parameters [56]. The paralogous gene pairs were used to calculate the synonymous (Ks) and nonsynonymous (Ka) values using the KaKs_Calculator2.0 [57]. Divergence time (T) was calculated using the formula T = Ks/2λ × 10 − 6(λ = 6.5 × 10^−9^ for grasses) million years ago (Mya) [58, 59].

### Prediction of cis-responsive elements on the promoters of *ZmMT* genes

The 2000 bp genomic regions upstream of the initiation codon (ATG) of *ZmMT* genes were obtained and used to search for the cis-acting regulatory elements in PlantCARE database and PLACE database (http://www.dna.affrc.go.jp/PLACE/). TF binding sites were obtained by searching PlantTFDB (http://planttfdb.cbi.pku.edu.cn/).

### Transcriptomic and quantitative real-time PCR (RT-qPCR) analysis

For spatial expression analysis *ZmMT* genes, transcriptomic sequencing (RNA-seq) data were collected from previous research [60]. These data represent 23 tissues of the spanning vegetative and reproductive stages of maize development. Gene transcript levels in various tissues were valued by fragments per kilobase (kb) of exon model per million mapped reads (FPKM) and the heat map was generated by using R (v3.4.0) with the log2 (FPKM + 1).

For the qRT-PCR assay, total RNA was extracted using the HiPure Universal RNA Kit (Biodata, Hefei, China) from respective tissues according to the manufacturer’s instructions. RNase-free DNase I (Biodata, Hefei, China) was used to remove genomic DNA. Total RNA (1 μg) was used to synthesize first-strand cDNAs using an equivalent amount of oligo-(dT)_15_ and random primers in 20 μL). volume with the GoScript™ Reverse Transcriptase 431 system (Promega, Madison, WI, USA) according to the manufacturer’s protocols. RT-qPCR was performed in 96-well plates in an ABI 7500 Real-time system (ABI, Alameda, CA, USA) using the SoAdvancedTM Universal SYBR® Green Supermix detection system (Bio-Rad, Hercules, CA, USA). The RT-qPCR reaction in a total volume of 10 μL consisted of 5 μL SYBR® Green Supermix, 0.5 μL of forward primer (10 μM), 0.5 μL of reverse primer (10 μM), 1 μL of cDNA, and 3 μL of ddH_2_O. The cycling conditions were 95 °C for 2 min, followed by 40 cycles at 95 °C for 15 s and 60 °C for 1 min. After 40 cycles, melting curve analysis was performed ranging from 60 to 95 °C. Maize 18S rRNA was used as the internal reference gene. The relative gene expression level was calculated using the 2^−ΔΔCq^ method. All RT-qPCR experiments were carried out using three biological replicates of each sample. The gene-specific primers used for RT-qPCR are listed in Additional file 1: Table S2.

## Results

### Genome-wide identification of *ZmMTs* in maize

A total of 9 *ZmMT* genes termed *ZmMT1* to *ZmMT9* were identified (Table 1, Additional file 1: Table S3) by searching the *Zea mays* genome using known MT encoding genes and *OsMt* genes as queries. Most of all the identified ZmMT proteins contained one highly conserved Metallothio_2 domain, while ZmMT4 proteins contained a Metallothio_PEC domain. The length of the amino acid sequence in the ZmMT proteins (Table 1) ranged from 75 (*ZmMT8*) to 84 (*ZmMT7*), with an average of 7.78 kDa protein molecular weight. Subcellular localization prediction by PSORT shown that five ZmMTs were located in the chloroplast (chlo) and four were located at mitochondrion (mito).

**Table 1:** Identified *ZmMT* genes from maize and their related information

Gene name	Gene ID	ORF (aa)	Subcellular Location of Protein	Protein Molecular Weight	GRAVY	Protein Isoelectric Point	Domain	Group
ZmMT1	Zm00001d008620	82	mito	7.90 kDa	− 0.182	pH 5.66	Metallothio_2	II
ZmMT2	Zm00001d011063	82	chlo	7.64 kDa	0.295	pH 4.26	Metallothio_2	II
ZmMT3	Zm00001d029546	79	mito	7.67 kDa	− 0.139	pH 4.91	Metallothio_2	I
ZmMT4	Zm00001d029778	77	mito	7.71 kDa	− 0.49	pH 7.37	Metallothio_PEC	IV
ZmMT5	Zm00001d035659	83	chlo	7.80 kDa	0.119	pH 4.67	Metallothio_2	II
ZmMT6	Zm00001d035662	80	chlo	7.59 kDa	0.1	pH 4.26	Metallothio_2	II
ZmMT7	Zm00001d039859	84	chlo	9.01 kDa	− 0.012	pH 8.26	Metallothio_2	II
ZmMT8	Zm00001d039914	75	mito	7.21 kDa	0.044	pH 6.04	Metallothio_2	II
ZmMT9	Zm00001d048611	76	chlo	7.52 kDa	− 0.171	pH 4.62	Metallothio_2	I

GRAVY(grand average of hydropathicity),Chloroplast (chlo),Mitochondrion (mito)

### Evolutionary relationships of *ZmMTs*

**Fig. 1:**
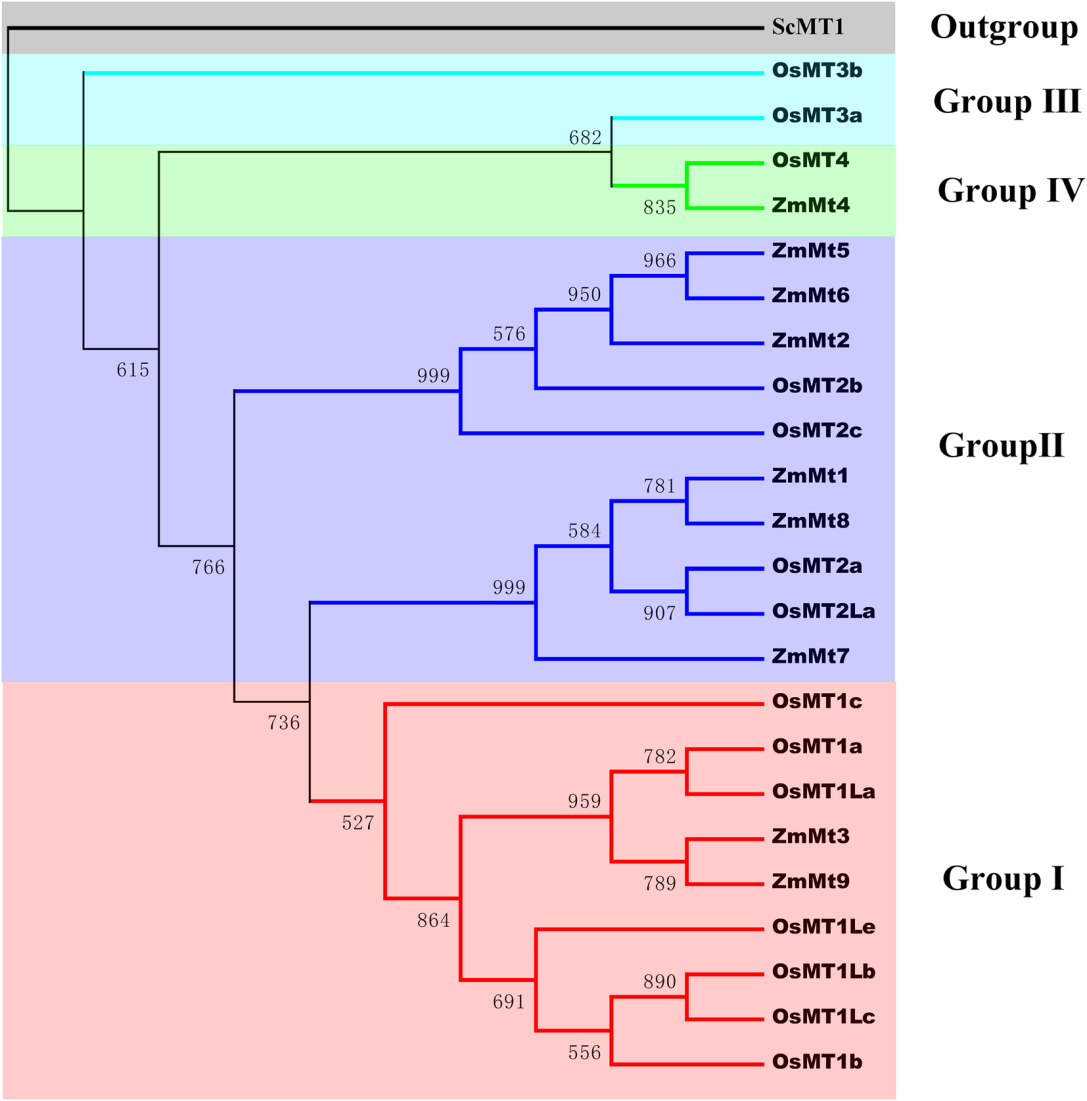
Classification of different groups of *ZmMTs*. Different color regions are used to distinguish different subgroups. The neighbor-joining (NJ) method was used to analyze the evolutionary trees of 9 *ZmMTs* and 14 *OsMTs*

The phylogenetic relationship among the nine identified ZmMT proteins was examined based on OsMt proteins from the four groups, and a rooted tree with ScMT1 as outgroup was built by ClustalX 2.1 (Fig. 1). All the ZmMT proteins were classified into three major groups (I, II and IV) (Table 1, Fig. 1). Two members of *ZmMTs* (*ZmMT3* and *ZmMT9*) belonged to group I, six members (*ZmMT1*, *ZmMT2*, *ZmMT5*, *ZmMT6*, *ZmMT7* and *ZmMT8*) belonged to group II, and one member (*ZmMT4*) belonged to group IV (Fig. 1).

### Genomic structure, conserved domain and motif of ZmMT proteins

**Fig. 2:**
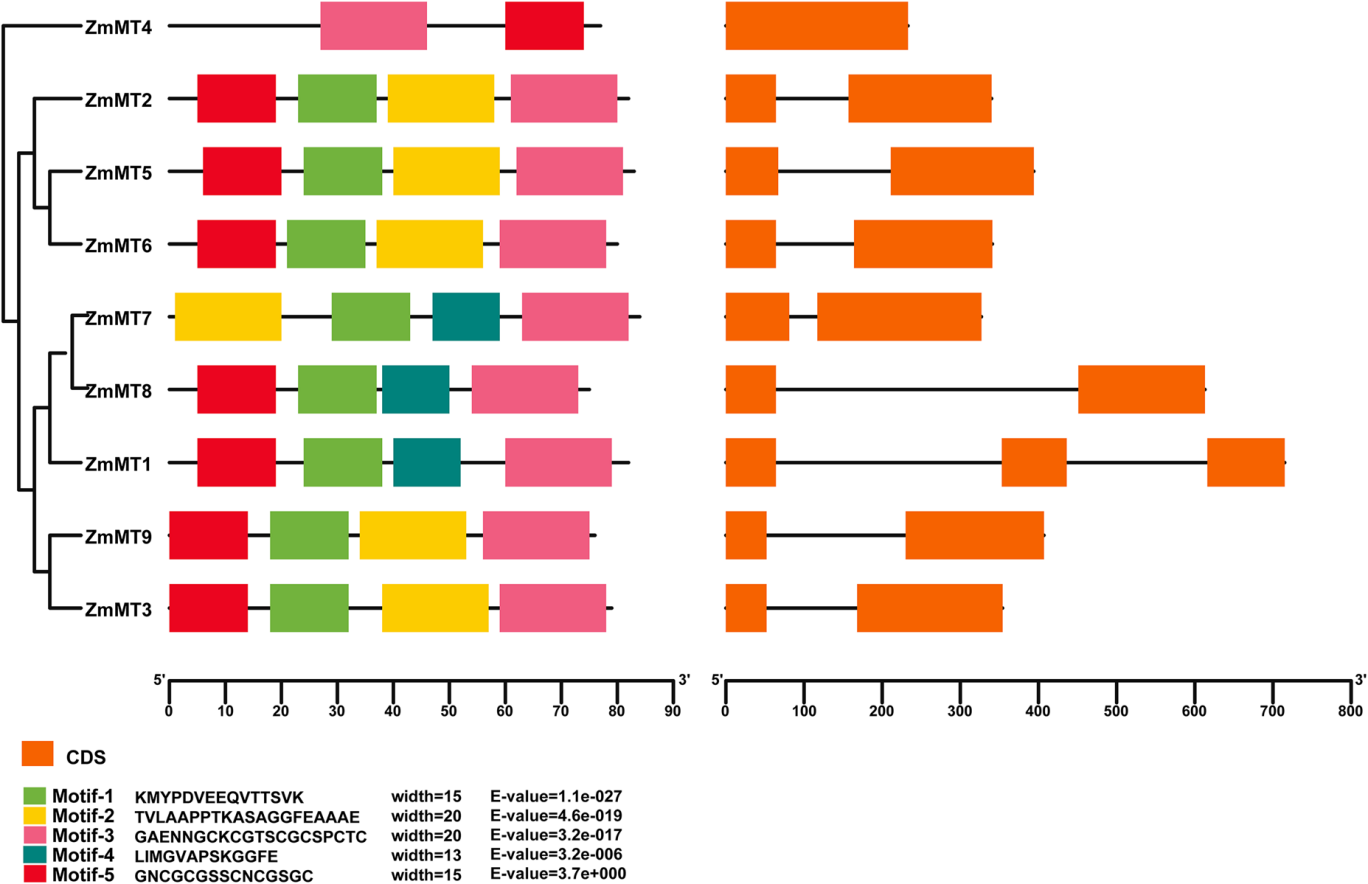
Conserved motifs and gene structures of *ZmMTs*. MEME analysis showing distributions of 5 conserved motifs and coding sequences (CDS) regions in 9 *ZmMTs* that were demonstrated with a colorful box

Through domain analysis, it can be seen that group I and group II members contain a Metallothio_2 domain and group IV members contain a Metallothio_PEC domain (Table 1). Genomic structure showed that *ZmMT4* has one exon, *ZmMT1* have 3 exons and other *ZmMTs* have 2 exons (Fig. 2).

The MEME program was used to predict the composition of the ZmMT proteins motifs. A total of five conserved motifs were detected (Fig. 2). Among them, motif 3 was conserved in all ZmMT proteins. Motif 1 and motif 5 were conserved in eight ZmMT proteins. Motif 2 and motif 4 were conserved in six and three ZmMT proteins. Except for motif 3 and motif 5, other motifs only exist in group I and group II. Interestingly, motif 3 exists in the 3' end of group I and group II, but in the middle of the sequence in group IV. In contrast, motif 5 exists in the 5' end of group I and group II, but in the 3' end in group IV (Fig. 2).

### Chromosomal location and cis-elements

**Fig. 3:**
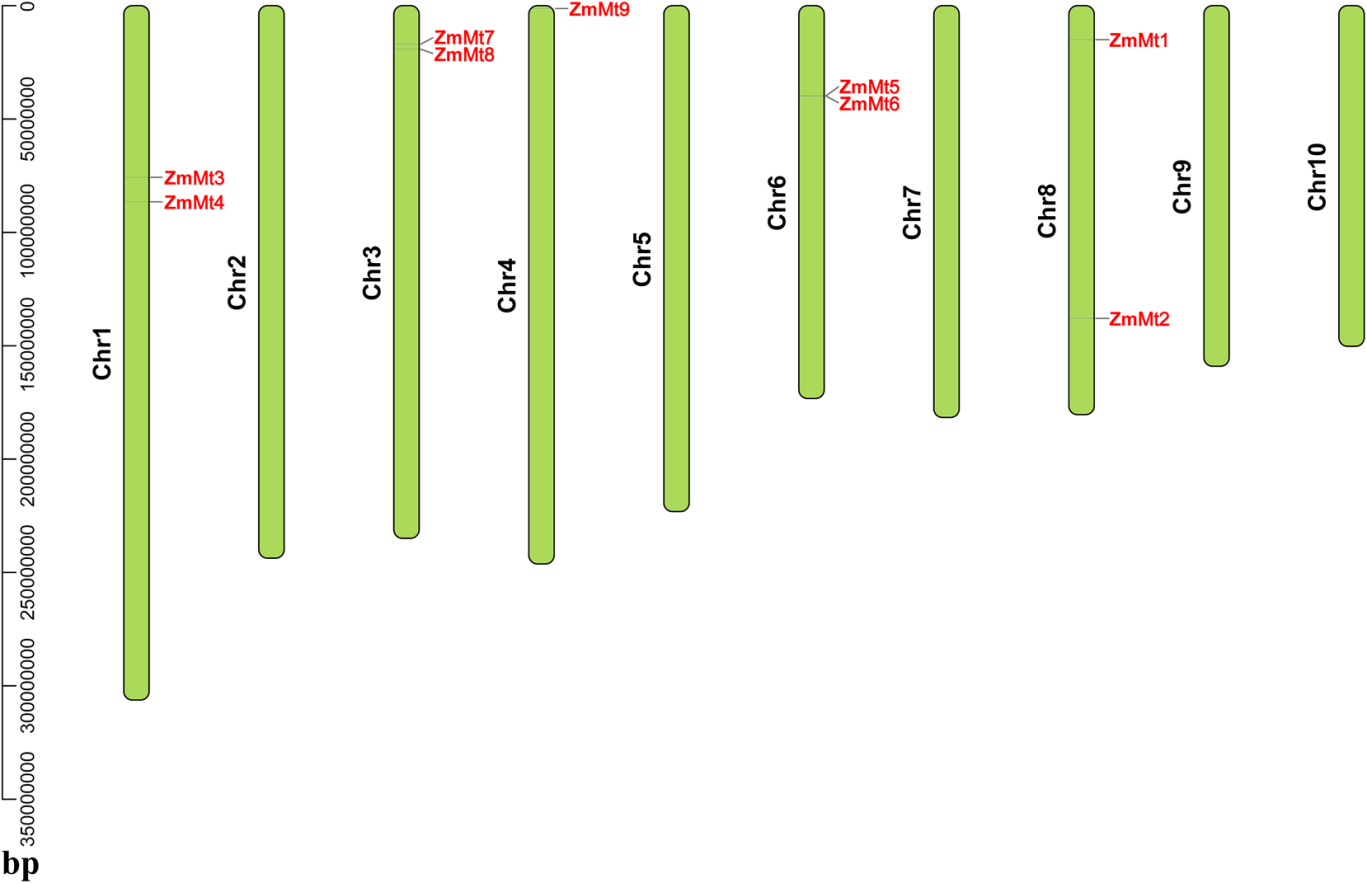
The chromosomal distribution of *ZmMT* genes

Analyses of the chromosomal distribution indicated that 9 *ZmMTs* were mapped on five chromosomes. *ZmMT9* was anchored on chromosome 4. *ZmMT1/ZmMT2*, *ZmMT3/ZmMT4*, *ZmMT5/ZmMT6*, *ZmMT7/ZmMT8* distributed on chromosome 8, 1, 6, and 3, respectively (Fig. 3). Based on the chromosomal distribution and paralogous analysis, the duplication events were proposed to occur in *Zea Mays* genome (Fig. 3 and Table 2). The substitution rate of nonsynonymous (Ka) and synonymous (Ks) is the basis for evaluating the positive selection pressure of duplication events, where Ka/Ks = 1 denoted neutral selection, Ka/Ks < 1 indicated purifying selection, and Ka/Ks > 1 referred to positive selection. KaKs Calculator 2.0 was used to calculate Ka/Ks of*ZmMTs* duplication event. Ka/Ks of paralogous ranged from 0.74 to 2.60. *ZmMT5/ZmMT6*, *ZmMT2/ZmMT5*, and *ZmMT2/ ZmMT6* had Ka/Ks > 1, that is, 2.60, 1.43, and 1.23, respectively. These results suggested that those*ZmMTs* were subjected to positive, negative, or balanced selection and functions of the duplicated genes became diverge along with the genome evolution after the duplication events of *ZmMTs*. The divergence time of the paralogous gene pairs of *ZmMT* gene pairs was estimated to be about range from 2.11 and 28.05 million years ago (Table 2).

**Table 2:** Paralogous and Ka/Ks analysis of the *ZmMT* gene pairs duplication

Seq_1	Seq_2	Ka	Ks	Ka/Ks	Divergence time (Mya)
*ZmMT5*	*ZmMT6*	0.07	0.03	2.60	2.11
*ZmMT2*	*ZmMT5*	0.14	0.10	1.43	7.45
*ZmMT2*	*ZmMT6*	0.14	0.12	1.23	8.97
*ZmMT1*	*ZmMT7*	0.23	0.23	0.99	17.78
*ZmMT7*	*ZmMT8*	0.27	0.36	0.74	28.05

The ratio of the number of nonsynonymous substitutions per nonsynonymous site (Ka) to the number of synonymous substitutions per synonymous site (Ks)

**Fig. 4:**
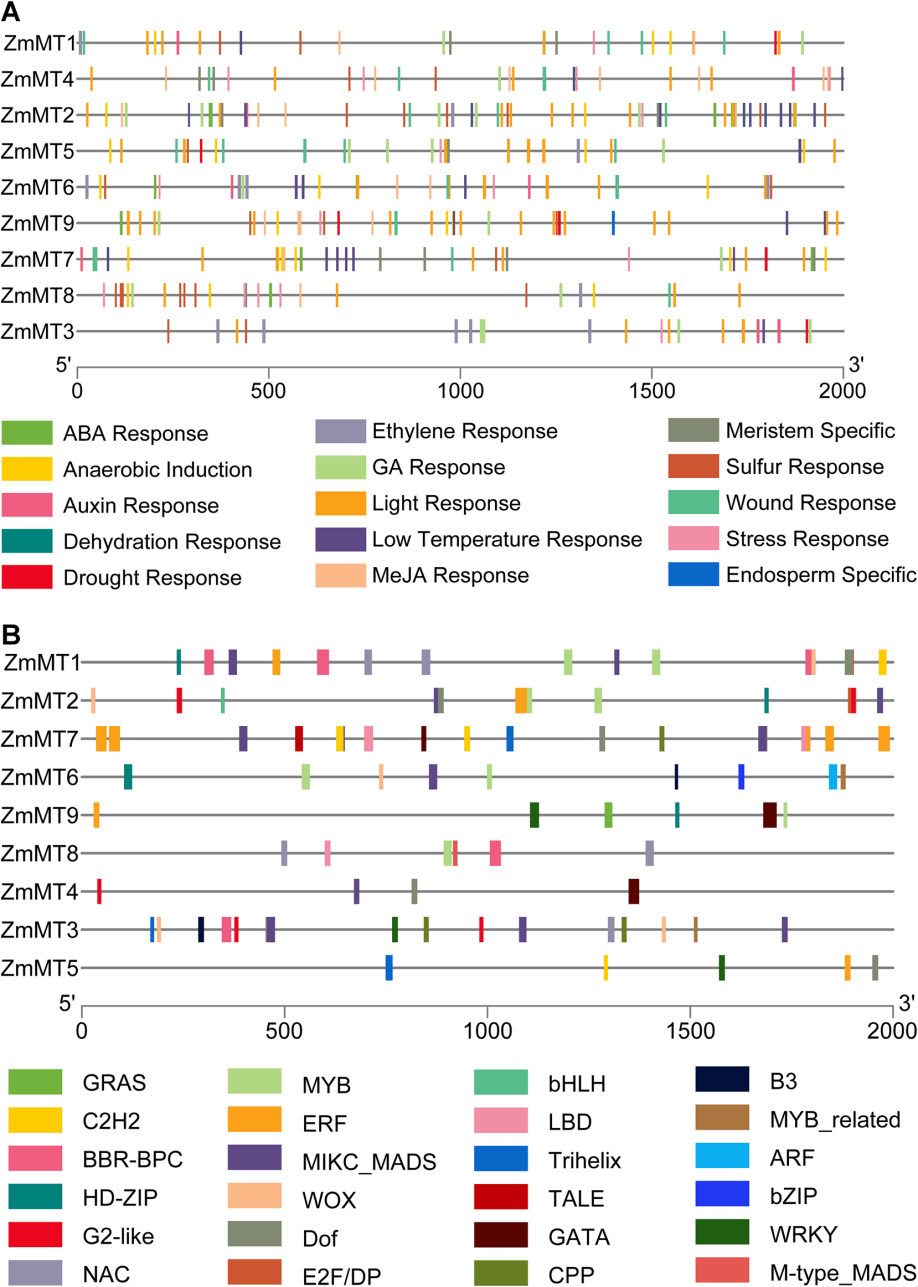
Prediction of cis-responsive elements (**A**) and transcription factor (**B**) binding sites in the 2-k upstream regulatory regions of *ZmMT* genes. **A** Prediction of cis-responsive elements in the 2-k upstream regulatory regions of *ZmMT* genes. Different cis-responsive elements are represented by different colored boxes. **B** Prediction of TF-binding sites in the 2-k upstream regulatory regions of *ZmMT* genes. Different TF-binding sites are represented by different colored boxes

To get an overview of the regulatory cis-acting elements involved in the responsiveness of abiotic and abiotic stresses, the 2 kb upstream sequences from each *ZmMTs* were programmed in PlantCARE and Plance server. As shown in Fig. 4A, potential environmental factor-related cis-regulatory elements were predicted to be correlated with ABA response, light response, and MeJA response which were most widely spread in promoters of *ZmMTs*. Stress-responsive elements were predicted to be mostly distributed in *ZmMT4* and *ZmMT8*. Furthermore, some transcription factor (TF) binding sites, were also predicted as shown in Fig. 4B. In which, ERF binding sites are enriched in *MzMT7* promoter, and MYB binding sites are enriched in *MzMT1*, *MzMT2,* and *MzMT6* promoter. These results suggest that ERF and MYB may play an important role in regulating *ZmMT* gene expression.

### Expression profiling of *ZmMT* genes in different tissues

**Fig. 5:**
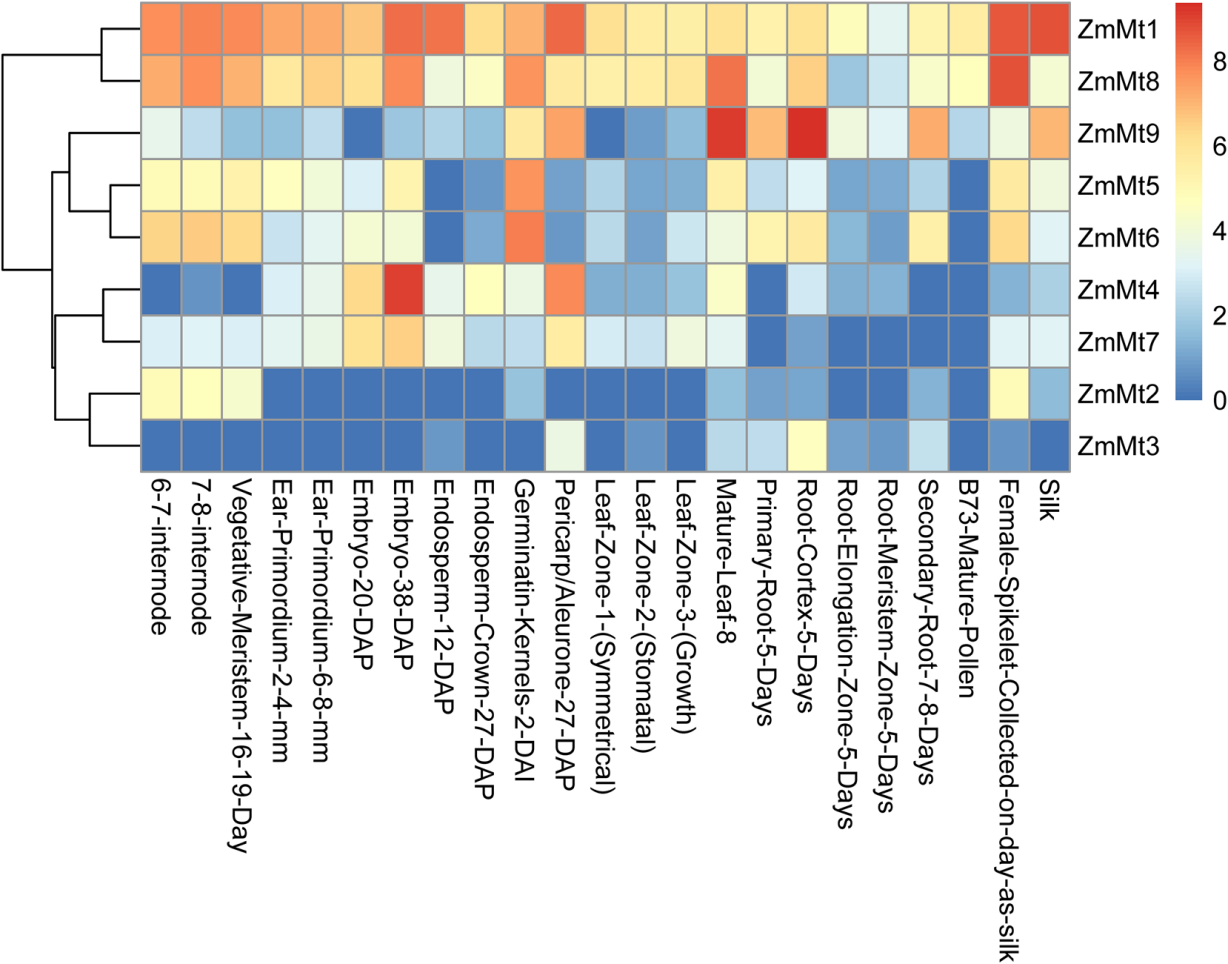
Hierarchical clustering of *ZmMT* genes expression levels in 23 tissues of the spanning vegetative and reproductive stages of maize development

To study the spatiotemporal specific expression of *ZmMT* genes, we used public available RNA-seq data of 23 tissues to investigate the expression profiles of the *ZmMT* genes. These data represent 23 tissues of the spanning vegetative and reproductive stages of maize development in Fig. 5. The cluster heatmap shows that *ZmMT1* and *ZmMT8* are highly expressed in all tissues. *ZmMT9* is highly expressed in germination kernels, pericarp, aleurone, mature leaf, primary root, root cortex, root elongation, root meristem zone, second root 7–8 days, female spikelet, silk. Both *ZmMT5* and *ZmMT6* are highly expressed in kernels. *ZmMT4* and *ZmMT7* are mainly expressed in seeds after fertilization, especially in the 38DAP embryo. *ZmMT2* and *ZmMT3* are expressed in low amounts in most tissues, among which *ZmMT2* is expressed in internodes and meristems higher than other tissues, and *ZmMT3* is only expressed relatively high in the root cortex.

### Response to heavy metal stresses

**Fig. 6:**
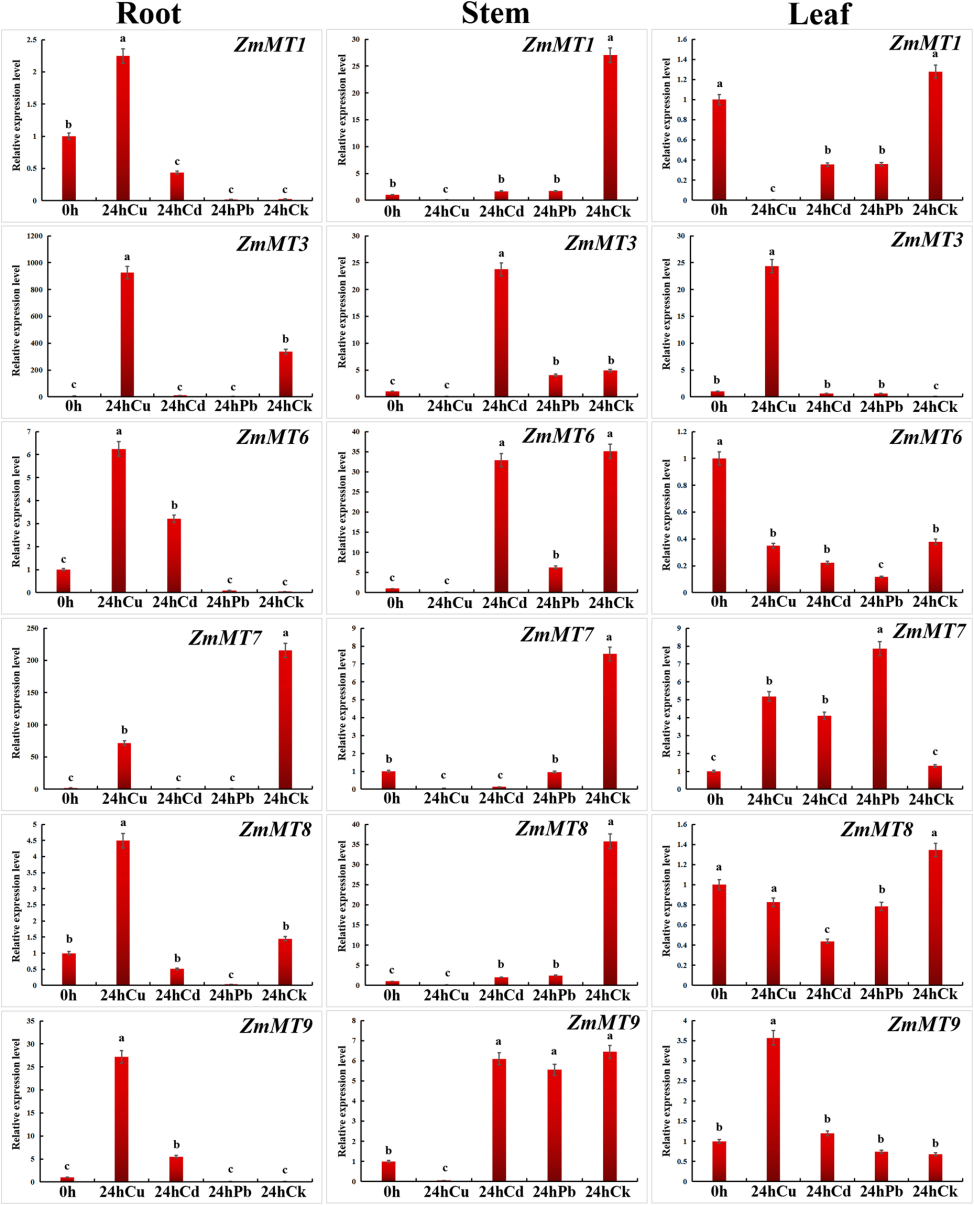
Expression profilings of *ZmMT* genes in different tissues

To verify the response expression of *ZmMTs*, the qRT-PCR assay was performed under three heavy metal stresses (Cu, Cd, and Pb). The expression of *ZmMT2*, *ZmM4,* and *ZmMT5* is not presented due to its extremely low signal (Fig. 6). In young roots, compared with CK, *ZmMT1*, *ZmMT3*, *ZmMT8*, *ZmMT9* are significantly up-regulated, while *ZmMT7* is significantly down-regulated under Cu stress. *ZmMT6* and *ZmMT9* are significantly up-regulated, while *ZmMT3* and *ZmMT7* are significantly down-regulated under Cd stress. *ZmMT3*, *ZmMT7*, and *ZmMT8* are significantly down-regulated under Pb stress. In young stems, compared with the control, *ZmMT1*, *ZmMT3*, *ZmMT6*, *ZmMT7*, *ZmMT8*, and *ZmMT9* are significantly down-regulated under Cu stress. *ZmMT3* is significantly up-regulated, while *ZmMT1*, *ZmMT7*, and *ZmMT8* are significantly down-regulated under Cd stress. *ZmMT1*, *ZmMT3*, *ZmMT6*, *ZmMT7*, *ZmMT8* are significantly down-regulated under Pb stress. In young leaves, compared with the control, *ZmMT3*, *ZmMT7*, *ZmMT9* are significantly up-regulated, while *ZmMT1* is significantly down-regulated under Cu stress. *ZmMT3*, *ZmMT7*, and *ZmMT9* are significantly up-regulated, while *ZmMT1* and *ZmMT8* are significantly down-regulated under Cd stress. *ZmMT3* and *ZmMT7* are significantly up-regulated, while CK, *ZmMT1*, *ZmMT6*, and *ZmMT8* are significantly down-regulated under Pb stress.

## Discussion

### Identification and structural conservation of ZmMT proteins

Maize is a widely cultivated cereal and tolerant to heavy metal ions-contaminated soils [61]. Maize has been used as an optimum plant for heavy metal ions phytoremediation in contaminated soils [62, 63]. Even if the entire genome sequencing of maize has been released for a decade, a thorough survey of *ZmMTs* has not been reported hitherto. We aimed to gain novel insights into the molecular aspects of the *ZmMT* gene family in maize. An initial comprehensive genome-wide analysis demonstrated that a total of 9 *ZmMTs* were identified from the recently released maize genome (Zm, B73_RefGen_v4) in Ensembl Plants.

The members of *MT* genes range from 4 (*Hevea brasiliensis*) to 14 (*Oryza sativa*) in higher plants in the previous reports [15, 16, 20, 23, 29, 33, 34, 64, 65]. It was showed that the number of *ZmMT* genes is not correlated with genome size but with genomic ploidy. For example, only four *MT* genes were identified in *H. brasiliensis*, which has a genome of 1460 megabases (Mb), while *O. sativa* has 14 members of *MT* genes and a moderate 420 Mb genome, and arabidopsis has 7 members of *MT* genes and a compact 135 Mb genome. In *Brassica*, three diploid species, *B. rapa* (AA, 2n = 20), *B. oleracea* (CC, 2n = 18), and *B. nigra* (BB, 2n = 20) have 8, 9 and 7 *MT* genes while in two allotetraploid species, *B. napus* (AACC, 2n = 38) and *B. juncea* (AABB, 2n = 36) have 16 and 12 *MT* genes. The number of *ZmMT* genes was smaller when considering its much larger genome size (~ 2500 Mb) [48] as compared with those of arabidopsis (125 Mb) [64] and rice (420 Mb) [65]. All ZmMTs proteins contain 75 to 84 amino acids, similar in length to the MTs of arabidopsis and rice [48, 64, 65].

As described in the results, the *ZmMT* genes in maize can be divided into three main groups based on phylogenetic analysis. There is no group III type gene in the maize genome, which is different from rice (Fig. 1). The motif-based sequence analysis tools (MEME) results showed that there are two conserved C-enriched motifs in the ZmMT protein sequences, namely motif 3 and motif 5. Motif 3 exists in all sequences, and motif 5 is deleted in the ZmMT7 protein sequence (Fig. 2). More interestingly, in most ZmMT proteins, motif 3 and motif 5 are present at the 3' and 5' ends of the protein, respectively. In ZmMT4 protein, motif 5 is present in the 5' segment, and motif 3 is present in the middle of the protein. The deletion of motif 5 and the misalignment of the two motifs may be caused by the rich transposon elements in the maize genome during genome replication evolution. In addition, motif1 and motif 2 exist in group I and group II, and motif4 only exists in group II.

### Cis-Elements of *ZmMT* genes

To analyze the regulatory elements of *ZmMT* genes, we extracted a 2 Kb candidate promoter sequence upstream of ATG and used the components of the PlantCARE database and the PLACE database to predict the regulatory elements on the promoter. The statistical results show that the promoter region of *ZmMT*genes contains a large number of hormone-related elements, such as ABA response, ethylene response, GA response, MeJA response components, light response components, low-temperature response components, and wound response components. Furthermore, using the PlantRegMap to analyze the binding elements on the promoter, the results showed that the promoters of *ZmMT2*, *ZmMT7,* and *ZmMT9* have a large number of ERF binding components, the promoters of *ZmMT2* and *ZmMT6* have a large number of MYB or MYB-related TF binding components, while the promoter of *ZmMT9* has a large number of NAC TF binding components, the promoter of *ZmMT7* has a large number of LBD binding components, the promoters of *ZmMT3*, *ZmMT5* and *ZmMT9* has a large number of WRKY binding elements. Hormones are able to decreasing levels of ROS or peroxidation for plants to against heavy metal stress[66]. Moreover, studies reported that hormone can enhance plant resistance to heavy metal stress[66]. For example, exogenous melatonin, epibrassinolide and jasmonic acid could enhance the antioxidant capacity of rice by inducing antioxidant enzyme activity, remove excess ROS, and thereby alleviate the toxicity of Cd and As to rice[67]. Thus, expressions of *ZmMTs* may regulate by hormones. These gave us a strong hint that molecular regulation of *ZmMTs* highly relies on the crosstalk among hormones, stress, and TFs (Fig. 3 and Additional file 1: Table S3). It also indicated that the regulation of *ZmMTs* by hormones may directly affect their promoters, or regulation is initiated indirectly by ERF, MYB, WRKY, or other TFs.

### Spatio-temporal expression and responses to heavy metal ions of *ZmMTs*

RNA-seq data of 23 tissues of the spanning vegetative and reproductive stages of maize development showed that *ZmMTs* have five expression patterns. Among them, *ZmMT1* and *ZmMT8* have higher expression in all tissues, indicating that these two genes may have important regulatory roles in the whole plant growth and development cycle of maize. *ZmMT9* is highly expressed in germination kernels, pericarp, aleurone, mature leaf, primary root, root cortex, root elongation, root meristem zone, second root 7–8 Days, spikelet, silk, indicating that *ZmMT9* may play an important regulatory role in the development of female organs, the development of root tissues and seed germination. Both *ZmMT5* and *ZmMT6* are highly expressed in kernels. *ZmMT4* and *ZmMT7* are mainly expressed in the seeds after fertilization, especially in the 38 DAP embryo, indicating that these two genes may be involved in the regulation of fertilization and seed maturity. *ZmMT2* and *ZmMT3* are expressed in low amounts in most tissues. *ZmMT2* is expressed in internodes and meristems higher than other tissues, indicating that *ZmMT2* may regulate the growth of aerial parts. *ZmMT3* is only expressed relatively high in root cortex, which shows that *ZmMT3* may be involved in regulating the growth and development of the underground part. Under different tissues and different heavy metal stress conditions, each gene has a different response pattern, indicating that plants have different regulatory mechanisms for different heavy metal stresses. In root, *ZmMT1, ZmMT6, ZmMT8* are all activated by Cu stress, however, *ZmMT6* is also sensitive to Cd stress. In stem, *ZmMT1* and *ZmMT8* are not activated by metal ion stressed. While in leaf, expression of *ZmMT1, ZmMT6, and ZmMT8* are suppressed by stress. But *ZmMT3, ZmMT7,* and *ZmMT9* are up-regulated in leaf under Cu stress. It is showed that most of *ZmMTs* in root are important for responding to heavy metal stresses, whereas the situation in stem and leaf are totally differed.

## Conclusions

MT proteins play an important role in the growth and development of plants and the regulation of stress response to heavy metals. The abundant regulatory elements and TF binding sites on the *MT* gene promoters result in a wide range of spatiotemporal expressions and different responses to heavy metal signals. Although the MT family genes have been identified and studied in different plants over the past two decades, the *MT* genes for maize has not been fully analyzed so far. Here, we provide bioinformatic analysis and quantitative analysis of expression level by genome-wide analysis of maize MT family. In summary, a large number of hormone regulatory elements and hormone-related transcription factor binding sites are resided on the *ZmMT* promoters. The specific expression of *ZmMTs* in different tissues of maize and the response to different heavy metal stresses are revealed that the role of MT in plant growth and development, and stress resistance to heavy metals.

## Supplementary Information

**Additional file 1: Table S1.** The detailed components of the nutrient solution. **Table S2.** Primers for qRT-PCR. **Table S3.** Identified *ZmMT*genes from maize and their related information.

## Data Availability

Please contact author for data requests.

## Abbreviations

MT: Metallothionein
TF: Transcription factor
chlo: Chloroplast
mito: Mitochondrion
Cd: Cadmium
Cu: Copper
Pb: Lead
GSH: Glutathione
PCs: Phytochelatins
